# Effect of Pulsed Electromagnetic Field on Bone Formation and Lipid Metabolism of Glucocorticoid-Induced Osteoporosis Rats through Canonical Wnt Signaling Pathway

**DOI:** 10.1155/2016/4927035

**Published:** 2016-01-28

**Authors:** Yuan Jiang, Hui Gou, Sanrong Wang, Jiang Zhu, Si Tian, Lehua Yu

**Affiliations:** Department of Rehabilitation Medicine and Physical Therapy, Second Affiliated Hospital, Chongqing Medical University, Chongqing 400010, China

## Abstract

Pulsed electromagnetic field (PEMF) has been suggested as a promising method alternative to drug-based therapies for treating osteoporosis (OP), but the role of PEMF in GIOP animal models still remains unknown. This study was performed to investigate the effect of PEMF on bone formation and lipid metabolism and further explored the several important components and targets of canonical Wnt signaling pathway in GIOP rats. After 12 weeks of intervention, bone mineral density (BMD) level of the whole body increased significantly, serum lipid levels decreased significantly, and trabeculae were thicker in GIOP rats of PEMF group. PEMF stimulation upregulated the mRNA and protein expression of Wnt10b, LRP5, *β*-catenin, OPG, and Runx2 and downregulated Axin2, PPAR-*γ*, C/EBP*α*, FABP4, and Dkk-1. The results of this study suggested that PEMF stimulation can prevent bone loss and improve lipid metabolism disorders in GIOP rats. Canonical Wnt signaling pathway plays an important role in bone formation and lipid metabolism during PEMF stimulation.

## 1. Introduction

Glucocorticoids (GCs) are frequently used to treat various diseases, such as bronchial asthma, rheumatoid arthritis, chronic renal diseases, collagen diseases, and pulmonary and skin diseases [1, 2]. Long-term use and (or) higher doses of GCs are inevitably in clinics due to the improvements in the outcome of these diseases. Nevertheless, approximately 30–50% of patients receiving long-term GCs therapy develop glucocorticoid-induced osteoporosis (GIOP) and osteonecrosis [3]. GIOP is the most frequently occurring type of secondary OP [4]. The risk of fractures in GIOP patients is characterized by closely correlating with the daily and cumulative doses of GCs, but it does not seem to correlate with the specific underlying disease [5]. Lipid metabolism disorders, specifically hyperlipidemia, are frequently accompanied by GIOP as well as postmenopausal OP. Lipid metabolism disorders are deemed to be a complication of GIOP [6] and it can exacerbate the degree of GIOP, because GCs induce adipocyte formation in bone marrow and further negatively affect the bone status. Unfortunately, the patients often have to tolerate the GIOP and hyperlipidemia during long-term GCs treatment because treatment options are limited [7]. Therefore, to choose an effective therapy of complications of GCs therapy is very important.

Currently, the clinical pharmacological treatments of GIOP rely on medications similar to the drugs which are used for the treatment of postmenopausal OP, including those with bisphosphonates, raloxifene, hormone replacement, parathyroid hormone (PTH), calcium, vitamin D, calcitonin, fluoride, testosterone, and anabolic steroids [7, 8]. These antiosteoporosis drugs also can modulate lipid metabolism in OP patients, but the risks of potential complications in long-term treatment cannot be ignored. For example, use of bisphosphonates is accompanied by some potential side effects, such as gastrointestinal complaints, osteonecrosis of the jaw, and atypical subtrochanteric or diaphyseal femoral fractures [9]. The prolonged use of hormone replacement is restricted because of potential complications such as breast cancer, uterine bleeding, and cardiovascular events [1]. Therefore, safe and noninvasive biophysical countermeasures for complications of GCs therapy might be more promising in clinical application.

Pulsed electromagnetic fields (PEMFs) have been suggested as promising method alternatives to drug-based therapies for treating a wide range of bone disorders, such as fresh fracture, delayed and nonunion fractures, diabetic osteopenia, osteoporosis, osteonecrosis, and osteoarthritis [10, 11]. PEMF is useful in enhancing BMD in OP patients and preventing bone loss in animal models of disuse OP, tail-suspension OP, ovariectomy-induced OP, and diabetes-mellitus-induced OP [12–14]. Several previous studies were using PEMF to prevent steroid-associated osteonecrosis and found that PEMF can improve serum lipid levels [15, 16].

Wingless-type MMTV integration site (Wnt) signaling pathway plays a critical role in maintenance of bone mass and is able to suppress adipogenesis and promote osteoblastogenesis of bone marrow mesenchymal stromal cells (BMSCs) [17]. PEMF stimulation can activate Wnt signaling pathway during treatment of bone loss in ovariectomized rats [18, 19]. However, the skeletal dynamics and pathogenesis of GIOP are distinctly different from other OP that associated with ageing, estrogen deprivation, and immobilization [5, 20]. Few studies have investigated the effects of PEMF on bone status, lipid metabolism, and related signaling pathway mechanisms in GIOP animal models. Particularly, GCs can directly induce differentiation of BMSCs into adipocytes and inhibit osteogenic differentiation through downregulating the Wnt signaling pathway [16, 21, 22]. In this present study, we established GIOP SD rats model by intramuscular injection with dexamethasone to investigate the effect of PEMF on bone formation and lipid metabolism and further explored the several important components and targets of canonical Wnt signaling pathway in GIOP rats.

## 2. Materials and Methods

### 2.1. Establishment of GIOP Rats Model

All animal experimental protocol and care were approved by the Institutional Animal Care and Use Committee of Chongqing Medical University. Totally, 40 female Sprague Dawley rats were employed, aged 3 months, weighing 210 ± 20 g and housed individually, and maintained under controlled environmental conditions (12-hour light-dark cycle, temperature 22°C with humidity of 50% ± 5%). All rats had unrestricted access to water and food. After one week of acclimatization, 10 rats were randomly divided into control groups. The rest of the rats were injected with dexamethasone sodium phosphate injection (DXMT, 2.5 mg/kg) into their right haunch muscles to establish GIOP rat models, twice a week, 12 weeks in a row. After 12 weeks, those rats received no DXMT intervention and were randomly divided into GIOP group (*n* = 10), calcium supplement group (calcium group, *n* = 10), and PEMF group (*n* = 10). The rats in the control group were injected with equivalent dose of normal saline into their right haunch muscles, twice a week, 12 weeks in a row. Subsequently, the fasting rats in each group received intraperitoneal anesthesia of 10% chloral hydrate (3.5 mL/kg). The rats were positioned on the operation platform in supine position. After the fur on the neck of rat was removed and skin was sterilized, a midline 1–1.5 cm longitudinal incision was performed. The left cephalic artery of rat was found to take blood (1.5–2 mL), and then the incision was stitched. Blood specimens were centrifuged to get serum. Bone mineral density (BMD) and bone mineral content (BMC) were measured by using dual energy X-ray absorptiometry (Lunar iDXA, GE Healthcare) equipped with dedicated software for small animal measurements, and serum biochemical analyses were operated to investigate whether the GIOP rat model was successfully induced by injecting DXMT and associated with lipid metabolism disorders.

### 2.2. GIOP Rats Treatments

The rats in calcium group received oral calcium (56.25 mg/kg, calcium gluconic tablets, Hainan Pharmaceutical Factory Co., Ltd., China), once a day, 12 weeks in a row. At the same time, the rats in control group, GIOP group, and PEMF group were perfused with the same volume of saline as the criteria of body mass. The rats in PEMF group were exposed to PEMF, which was generated by the ZH-21 osteoporosis treatment system (Chongqing Zhonghuan Electronic Technology Co., Ltd., China) with a frequency of 50 Hz and an intensity of 4.0 mT, once a day, 40-minute treatment every day, 12 weeks in a row. The waveform is square wave with pulse width 200 *μ*s. At the same time, the rats in control group, calcium group, and GIOP group were also placed in the ZH-21 osteoporosis treatment system, but the treatment system was not running to provide sham PEMF stimulation.

### 2.3. Bone Mineral Density and Bone Mineral Content Measurement

After a period of 12 weeks' treatment, all rats received intraperitoneal anesthesia of 10% chloral hydrate (3.5 mL/kg). Then the rats were positioned on the platform of dual energy X-ray absorptiometry (Lunar iDXA, GE Healthcare) in prone position. The BMD and BMC of head, upper limb, femur, trunk, rib, pelvis, spine, and the whole body were detected and recorded.

### 2.4. Serum Biochemical Analysis

The fasting rats in each group received intraperitoneal anesthesia with 10% chloral hydrate (3.5 mL/kg). Then the rats were positioned on the operation platform before they were executed. The fur on the abdomen was removed and skin was sterilized; a midline 4-5 cm longitudinal incision was performed. The blood specimens were withdrawn from aorta abdominalis and then centrifuged to get serum. The serum calcium (Ca), phosphorus (P), alkaline phosphatase (ALP), triglyceride (TG), total cholesterol (TCHO), high density lipoprotein cholesterol (HDL), and low density lipoprotein cholesterol (LDL) were determined by automatic biochemical analyzer (TBA-120FR, Toshiba). The serum tartrate resistant acid phosphatase (TRAP) was determined by tartrate resistant acid phosphatase assay kit (Beyotime, China) according to the protocol of the manufacturer.

### 2.5. Histomorphometrical and Histopathological Analysis

After all rats were executed, the fourth lumbar (L4) vertebral bodies were collected. Each fourth lumbar was carefully cleaned and then decalcified by EDTA decalcifying solution (BOSTER, China) for 6 weeks. The vertebral body samples were put into the optimum cutting temperature compound (O.C.T. compound, Sakura) and quick freezing, then flat-placed in the cryostat mould, and cut into serial sections with cryostat (15 *μ*m per section). The serial sections were stained with Safranin-O/Fast green for histomorphometrical analysis and stained with hematoxylin-eosin (HE) staining solution for histopathological analysis. Finally, the trabecular bone structure of L4 vertebral bodies in each group was observed by inverted microscope. Histomorphometrical parameters were quantified by using the Image-Pro Plus 6.0 software (Media Cybernetics). The static parameters are calculated using the following formula [19]: percentage of trabecular area (Tb.Ar) = trabecular area (Tb.Ar)/bone area (T.Ar) × 100%; trabecular width (Tb.Wi) = (2000/1.199) × (Tb.Ar/trabecular perimeter [Tb.Pm]); trabecular number (Tb.N) = (1.199/2) × (Tb.Pm/T.Ar); trabecular separation (Tb.Sp) = (2000/1.199) × (T.Ar − Tb.Ar)/Tb.Pm.

### 2.6. Real-Time PCR Analysis

After all rats were executed, the right thighbones were collected. Total RNA was extracted from caput femoris using the TRIzol reagent (Beyotime, China) according to the manufacturer's instruction and quantified by spectrophotometry at a wavelength of 260 nm. Reverse transcription actions and PCR were performed using reverse transcriptase, oligo (DT) primers, and Taq DNA polymerase. The specific sequences of the primers for Wnt10b, low density lipoprotein receptor-related protein 5 (LRP5), *β*-catenin, axis inhibition protein 2 (Axin2), osteoprotegerin (OPG), receptor activator of nuclear factor *κ*B ligand (RANKL), dickkopf1 (Dkk-1), Sclerostin (SOST), Runt-related transcription factor 2 (Runx2), peroxisome proliferator-activated receptor-*γ* (PPAR-*γ*), CCAAT/enhancer binding protein-*α* (C/EBP*α*), and fatty acid binding protein 4 (FABP4) are listed in Table 1. GAPDH was used as an internal control. Relative mRNA expressions were defined as the ratio of target genes expression to GAPDH expression.

### 2.7. Western Blot Analysis

The caput femoris samples were milled and lysed with RIPA lysis buffer (Beyotime, China) for extracting total proteins. Protein samples were separated by sodium dodecyl sulfate-polyacrylamide gel electrophoresis and electrotransferred onto a polyvinylidene difluoride (PVDF) membrane. The PVDF membrane was blocked for 2 h at room temperature in TBS-Tween 20 (TBST) buffer containing 5% BSA, washed with TBST three times, and incubated overnight at 4°C with 1/500 dilution of Wnt10b antibodies, LRP5 antibodies, *β*-catenin antibodies, Axin2 antibodies, OPG antibodies, RANKL antibodies, Dkk-1 antibodies, SOST antibodies, Runx2 antibodies, PPAR-*γ* antibodies, C/EBP*α* antibodies, FABP4 antibodies (all purchased from Santa Cruz Biotech), and GAPDH antibody (1 : 1000, BOSTER, China), respectively. After being washed with TBST, the membranes were incubated with the secondary biotin-conjugated antibody and then with anti-biotin horseradish peroxidase- (HRP-) linked antibody (1 : 1000). Protein signals were detected using SuperSignal West Pico Chemiluminescent Substrate Trial kit (Thermo Scientific) and quantified by densitometry using Quantity One software (Bio-Rad).

### 2.8. Statistical Analysis

The statistical analysis was conducted using SPSS 20.0 for Windows software. Data were presented as mean ± standard deviation (SD). Differences in group were analyzed by using repeated measure analysis of variance (ANOVA). Intergroup comparisons were performed using the least significant difference (LSD) test for multiple comparisons. *P* < 0.05 was considered to be statistical significance.

## 3. Results

### 3.1. GIOP Rats Model

**Figure 1:**
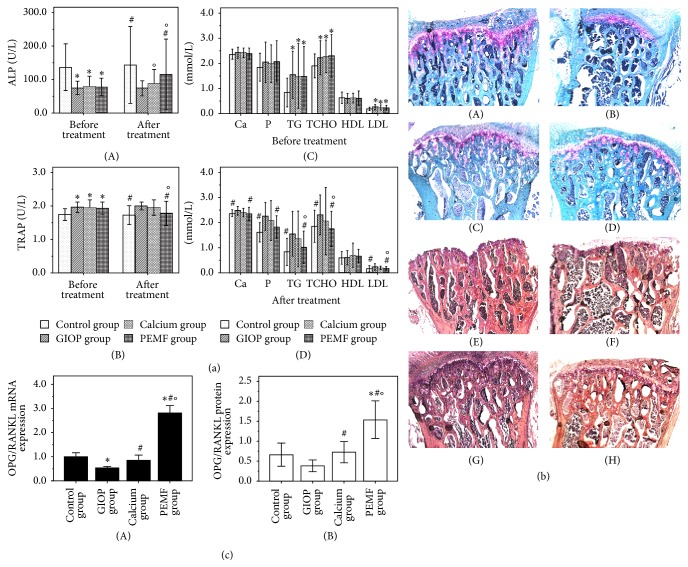
The serum concentrations of alkaline phosphatase (ALP) and tartrate resistant acid phosphatase (TRAP) in each group before and after treatment ((a) A, B). The results of serum biochemical analysis in each group before and after treatment include serum calcium (Ca), phosphorus (P), triglyceride (TG), total cholesterol (TCHO), high density lipoprotein cholesterol (HDL), and low density lipoprotein cholesterol (LDL) ((a) C, D). The L4 vertebral bodies were stained with Safranin-O/Fast green: ((b) A) control group, ((b) B) GIOP group, ((b) C) calcium group, and ((b) D) PEMF group; bone tissue was stained in grayish-green or blue; cartilage tissue was stained in red. The L4 vertebral bodies also were stained with HE staining solution: ((b) E) control group, ((b) F) GIOP group, ((b) G) calcium group, and ((b) H) PEMF group. The rate of OPG to RANKL mRNA and protein expressions in each group after treatment ((c) A, B). Compared with the control group, ^*∗*^ *P* < 0.05; compared with the GIOP group, ^#^ *P* < 0.05; compared with calcium group, ^O^ *P* < 0.05.

After a period of 12 weeks with DXMT or normal saline intervention, the rats in each group were detected by dual energy X-ray absorptiometry. The results of BMD are shown in Table 2. Compared with the normal rats in control group, the BMD values of head, femur, trunk, rib, pelvis, spine, and whole body of GIOP rats models in GIOP group, calcium group, and PEMF group were significantly declined, respectively (all *P* < 0.05). As shown in Table 3, the BMC values of trunk, rib, pelvis, spine, and whole body in GIOP rats models were significantly lower than those in the normal rats (*P* < 0.05). The serum ALP level of the GIOP rats models was lower and the serum TRAP level was higher, compared to the normal rats (Figure 1(a)(A, B)). In contrast with the control group, the serum Ca and phosphorus level of GIOP group, calcium group, and PEMF group had increased, but there was no significant difference among the four groups. On the contrary, the serum TG, TCHO, and LDL of GIOP rats models in GIOP group, calcium group, and PEMF group were significantly higher than normal rats in control group (Figure 1(a)(C)). All the abovementioned results demonstrated that the GIOP rats model was successfully established, and disorder of lipid metabolism (hyperlipemia) was accompanied by bone mass loss.

### 3.2. Body Weight

Rats were weighed every week. Before PEMF stimulation or oral calcium treatment, the body weights of rats in control group, GIOP group, calcium group, and PEMF group were 266.2 ± 19.2 g, 260.1 ± 26.1 g, 259.6 ± 14.2 g, and 261.8 ± 10.4 g. There was no significant difference in body weight among the four groups before PEMF stimulation or oral calcium treatment (*P* > 0.05). After 12 weeks of different treatment, the body weights of rats in control group, GIOP group, calcium group, and PEMF group were 294.1 ± 17.9 g, 281.6 ± 23.5 g, 283.7 ± 10.6 g, and 285.9 ± 8.7 g. There was no significant difference in body weight among the four groups after applying different treatment methods (*P* > 0.05).

### 3.3. Bone Mineral Density and Bone Mineral Content Measurement

As shown in Table 2, after 12 weeks of PEMF treatment, the BMD values of femur, trunk, pelvis, spine, and the whole body in PEMF group were significantly higher than those before treatment (*P* < 0.05), and the BMD values of spine and the whole body were significantly higher than those in GIOP group (*P* < 0.05). The BMC values of head, rib, and the whole body were significantly higher than those before treatment (*P* < 0.05). After 12 weeks of calcium supplement treatment, the BMD values of pelvis and spine in calcium group were significantly higher than those before treatment (*P* < 0.05) and only the BMD value of spine was significantly higher than that in GIOP group (*P* < 0.05). The BMC value of pelvis and the whole body was significantly higher than that before treatment (*P* < 0.05).

### 3.4. Serum Biochemical Analysis

As shown in Figure 1(a), the serum ALP level of PEMF group after treatment was significantly higher than before treatment and GIOP group, respectively (*P* < 0.05). The serum ALP level of GIOP group was still lower than control group after 12 weeks of sham treatment period free of DXMT (*P* < 0.05). The serum TRAP level of PEMF group after treatment was significantly lower than before treatment and GIOP group, respectively (*P* < 0.05). There was no significant difference in the serum TRAP level between the GIOP group and control group after sham treatment (*P* > 0.05). In contrast to GIOP group, the serum Ca and phosphorus level of calcium group and PEMF group have declined after 12 weeks of intervention treatment, while only phosphorus level of PEMF group had a significant reduction in contrast to before treatment (*P* < 0.05). The levels of serum TG and LDL of GIOP group were still lower than control group after 12 weeks of sham treatment period free of DXMT (*P* < 0.05). The level of serum TG, TCHO, and LDL of PEMF group after treatment had a significant reduction in contrast to before treatment and GIOP group, respectively (*P* < 0.05). However, the abovementioned blood lipids index in calcium group after treatment had decreased tendency, but there was no statistical difference after compared with before treatment and GIOP group, respectively (*P* > 0.05).

### 3.5. Histomorphometrical and Histopathological Analysis

After 12 weeks of different intervention treatment, the L4 vertebral bodies of four groups were stained with Safranin-O/Fast green for histomorphometrical analysis and shown in Figure 1(b)(A–D). Bone tissue was stained in grayish-green or blue and cartilage tissue was stained in red. The results of histomorphometrical analysis were shown in Table 4. Tb.Ar, Tb.N significantly declined and Tb.Sp significantly increased in GIOP group in contrast with normal rats in control group (*P* < 0.05). By contrast, PEMF stimulation and calcium supplement increased Tb.Ar, Tb.N and decreased Tb.Sp. The L4 vertebral bodies were stained with HE staining solution for histopathological analysis and shown in Figure 1(b)(E–H). In contrast with control group, the trabeculae were thinner and sparse, and cracks and breaks were observed in GIOP group. The trabeculae number in PEMF group and calcium group was slightly increased, and the trabeculae were thicker than GIOP group.

### 3.6. Real-Time PCR Analysis

**Figure 2:**
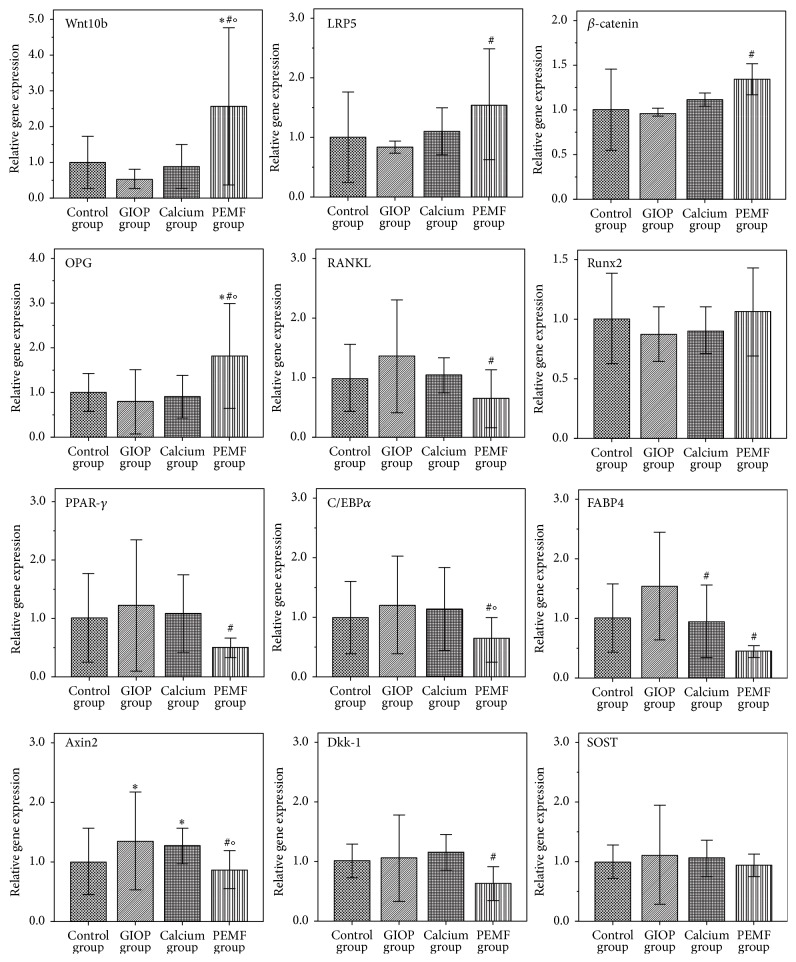
The relative mRNA expressions of target genes were estimated using real-time PCR analysis after 12-week interventions. Compared with the control group, ^*∗*^ *P* < 0.05; compared with the GIOP group, ^#^ *P* < 0.05; compared with calcium group, ^O^ *P* < 0.05.

The relative mRNA expressions of target genes were estimated using real-time PCR analysis. As shown in Figure 2, in contrast with GIOP group, the mRNA expressions of Wnt10b, LRP5, *β*-catenin, and OPG were significantly increased (all *P* < 0.05) and the mRNA expressions of Axin2, RANKL, PPAR-*γ*, C/EBP*α*, FABP4, and Dkk-1 were significantly decreased (all *P* < 0.05) in PEMF group after 12 weeks of PEMF stimulation. Only the mRNA expression of FABP4 was significantly decreased (*P* < 0.05) in calcium group after 12 weeks of calcium supplement treatment. Moreover, the rate of OPG/RANKL mRNA expression level was significantly increased in PEMF group compared to the other three groups (*P* < 0.05, Figure 1(c)(A)).

### 3.7. Western Blot Analysis

**Figure 3:**
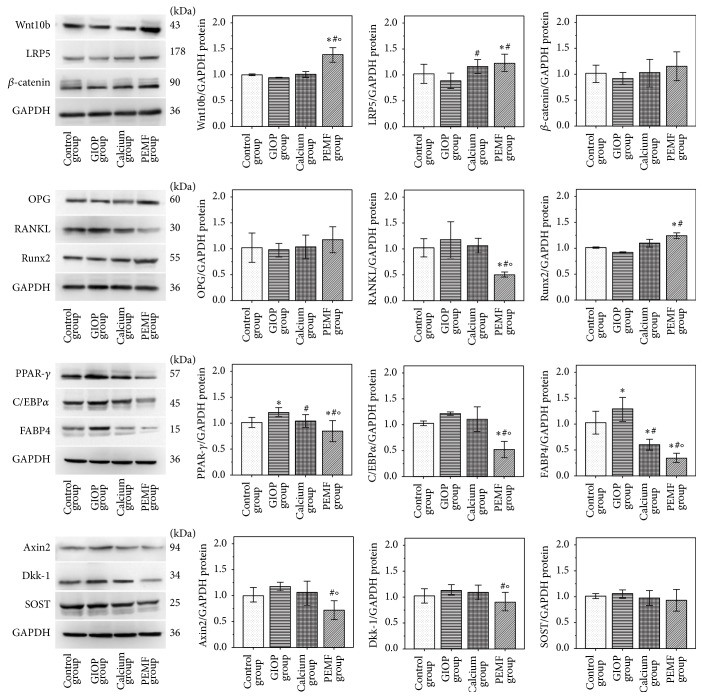
The protein expressions of target genes were estimated using Western blot analysis after 12-week interventions. Compared with the control group, ^*∗*^ *P* < 0.05; compared with the GIOP group, ^#^ *P* < 0.05; compared with calcium group, ^O^ *P* < 0.05.

The protein expressions of target genes were estimated using Western blot analysis. As shown in Figure 3, the protein expressions of PPAR-*γ* and FABP4 were significantly increased (all *P* < 0.05) in GIOP group compared with control group after 12 weeks of sham treatment period free of DXMT. In contrast with GIOP group, the protein expressions of Wnt10b, LRP5, and Runx2 were significantly increased (*P* < 0.05) and the protein expressions of Axin2, RANKL, PPAR-*γ*, C/EBP*α*, FABP4, and Dkk-1 were significantly decreased in PEMF group (*P* < 0.05). Only the protein expression of FABP4 was significantly decreased (*P* < 0.05) in calcium group. Moreover, the rate of OPG/RANKL protein expression level was significantly increased in PEMF group compared to the other three groups (*P* < 0.05, Figure 1(c)(B)).

## 4. Discussion

It is necessary to search for a suitable therapeutic method for GIOP with minor side effects and lower cost, due to the serious side effects and/or high cost of currently available therapies. PEMF is a safe and effective method of treating postmenopausal OP and steroid-associated osteonecrosis. At present, few studies have investigated the effects of PEMF on GIOP animal models or GIOP patients. The role of PEMF in GIOP needs further investigation. The laboratory rat is the preferred animal for most research due to the similarities in pathophysiologic responses between the human and rat skeleton [23]. Moreover, rat can be used for building the reliable animal model of GCs-induced osteopenia/osteoporosis and massive formation of adipocytes and attempting to mimic the bone changes seen in humans [6, 24]. Certainly, the phenotypes of GIOP rats depend on the age and dosage and the period of GC administration [25]. In this study, we chose female SD rats aged 3 months as the animal model. Dexamethasone was given to rats by intramuscular injection, because dexamethasone causes more skeletal complications than prednisone [23]. The detection results of BMC, BMD, serum ALP, serum TRAP, and serum lipid levels suggested that intramuscular injection with DXMT (2.5 mg/kg, twice a week) for 12 weeks can induce the GIOP and be accompanied by hyperlipidemia in experimental rats. BMD measurement is considered a testing standard for diagnosis of OP and often used to evaluate BMD and BMC in animal models [12, 26]. Dual energy X-ray absorptiometry (Lunar iDXA, GE Healthcare) with small animal software can be used to measure both total and regional BMD and BMC in rat. Many studies have shown GCs administration in humans increases the risk of skeletal fractures, particularly in the ribs and spine, which are mainly composed of trabecular bone [27, 28]. In this study, the BMD values of ribs and spine in GIOP rats were significantly lower than normal rats. Those results further demonstrated that GIOP rats can mimic the bone changes seen in GIOP patients. In addition, we found that the osteoporosis degree of GIOP rats has not improved dramatically after 12 weeks of sham treatment period free of DXMT. We suspect that GIOP rats find it hard to recover bone mass loss on their own steam if without any effective treatment method. By contrast, PEMF stimulation for 12 weeks increased the values of BMD and BMC of GIOP rats effectively, and the curative effect of calcium supplement treatment was less marked than PEMF stimulation. The trabecular bone microarchitecture is generally considered as a good predictor of bone mass loss and bone structure deterioration [29], and bone loss in GIOP is most obvious in trabecular bone structural changes [28]. In this study, histopathological analysis showed GCs caused thinning of trabeculae and deteriorated architecture of trabecular bone, suggesting that the trabecular bone structural changes of GIOP rat have not significantly improved after DXMT injection was stopped. However, PEMF stimulation improved the changes of trabecular bone as well as calcium supplement treatment after 12-week interventions. Histomorphometrical analysis shows that PEMF stimulation increased trabecular width and trabecular number. The results of the abovementioned analysis indicated that PEMF stimulation markedly improved the bone loss in GIOP rats.

ALP is a marker of early stage of osteoblast differentiation, and it is known to be importantly involved in the regulation of osteoblastic cell differentiation, proliferation, and migration during bone formation [8, 30]. TRAP is a marker of osteoclast activity, and it is used to measure the changes in bone resorption. In this study, PEMF stimulation significantly improved serum ALP level and reduced serum TRAP level after 12-week interventions, suggesting that PEMF can activate the osteoblast differentiation and bone formation; meanwhile, it can inhibit osteoclast function and bone resorption. TG, TCHO, LDL, and HDL are the commonly observed parameter to measure lipid metabolism in clinical practice [3]. Compared with normal rats, GIOP rats are characterized by increased levels of serum TG, TCHO, and LDL in this study, suggesting that long-term DXMT administration causes lipid metabolism disorders. Moreover, the levels of serum TG, TCHO, and LDL have not significantly improved after DXMT injection was stopped. However, PEMF stimulation significantly reduced levels of serum TG, TCHO, and LDL after 12-week interventions, suggesting that PEMF improved the lipid metabolism disorders, and the improvement effect was superior to calcium supplement treatment in GIOP rats.

In order to clarify the mechanism of PEMF stimulation, further experiments were in progress to evaluate the role of canonical Wnt signaling pathway in bone formation and lipid metabolism in GIOP rats. The maintenance of bone mass is determined by bone remodeling activity, which is characterized by a dynamic balance between osteoblastic bone formation and osteoclastic bone resorption [31]. Therefore, regulation of the functions of osteoblasts and osteoclasts is essential for the maintenance of bone mass. Osteoblasts and osteoclasts are differentiated from bone marrow mesenchymal stem cells (BMSCs). Activation of canonical Wnt signaling pathway promotes the differentiation of BMSCs into mature osteoblasts, suppresses the apoptosis of osteoblasts, and enhances the proliferation and mineralization of osteoblasts [11]. Wnt10b, LRP5, and *β*-catenin are a key link of canonical Wnt signaling pathway. Wnt10b is a positive modulator of bone formation, and it is expressed in bone marrow. The levels of Wnt10b are directly correlated with bone density and indirectly related to marrow adiposity [32]. LRP5 is a critical coreceptor for Wnt signaling pathway and upstream of *β*-catenin, and it plays an important role in skeletal development and bone maintenance [30]. *β*-catenin is an essential mediator of signals emanating from LRP5, and it can promote the survival and differentiation of osteoblasts [30]. In this study, the mRNA and protein expressions of Wnt10b, LRP5, and *β*-catenin were significantly increased in the GIOP rats after PEMF stimulation for 12 weeks, suggesting that canonical Wnt signaling pathway was activated during PEMF stimulation, which is in agreement with previous reports [16, 18]. RANK/RANKL/OPG signaling pathway plays a key role in differentiation and functional activation of osteoclasts [10, 12]. OPG and RANKL are mainly secreted by osteoblasts. OPG is a decoy receptor for the RANKL, and it prevents RANKL specifically from binding with RANK to promote osteoclast differentiation and activation [11]. Osteoclast activity is likely to depend on the relative balance of OPG and RANKL, and the OPG/RANKL ratio is an essential factor in bone resorption [30]. The OPG/RANKL ratio in PEMF group was significantly higher than GIOP group, suggesting that PEMF stimulation can promote the OPG/RANKL ratio for regulating osteoclast differentiation and preventing bone resorption. Canonical Wnt signaling pathway increases OPG secretion which is likely to depend on activation of *β*-catenin. *β*-catenin can upregulate OPG expression and increases the OPG/RANKL ratio in osteoblasts [32]. Spencer et al. [33] suggested that RANKL is a direct target of canonical Wnt signaling pathway. Taken together, different from the research results obtained before [12, 18, 19], we speculated that activation of canonical Wnt signaling pathway can promote RANK/RANKL/OPG signaling pathway during PEMF stimulation and further regulate the dynamic balance between osteoblastic bone formation and osteoclastic bone resorption in GIOP rats.

BMSCs can differentiate into osteoblasts, adipocytes, myocytes, and chondrocytes [34]. Excessive use of GCs can disturb lipid metabolism homeostasis directly by GCs inducing BMSCs differentiation into adipocytes. GCs also can upregulate the expression of PPAR-*γ*, downregulate the Runx2 to break the dynamic balance between adipogenesis and osteogenesis of BMSCs, and lead to fat tissue accumulation in bone marrow; eventually the degree of osteoporosis was increased [7, 15]. PPAR-*γ* is an adipogenic gene, and it plays a pivotal role in the regulation of adipogenesis and lipid metabolism homeostasis in synergy with another key adipogenic transcription factor C/EBP*α*. PPAR-*γ* and C/EBP*α* positively activate the transcription of each other [17]. FABP4 is a marker of mature adipocyte. On the contrary, Runx2 is an osteogenic gene, and it is a master regulator of BMSCs differentiation into osteoblast and RANKL expression in osteoblasts [35]. Furthermore, Runx2 is a transcriptional target of canonical Wnt signaling pathway and it involves negative regulation of PPAR-*γ* expression [10]. The inhibition of Runx2 by GCs is a critical mechanism of GIOP [36]. In this study, we found that PEMF stimulation significantly declined the mRNA and protein expressions of PPAR-*γ*, C/EBP*α*, and FABP4 and significantly increased the mRNA and protein expressions of Runx2. Canonical Wnt signaling pathway can suppress PPAR-*γ* and C/EBP*α* expression and upregulate the expression of Runx2 [6]. Axin2, a master scaffolding protein, is an intracellular inhibitor of canonical Wnt signaling pathway, and it is likely to involve positive regulation of PPAR-*γ* expression. Naito et al. [37] suggested that DEX promoted adipocyte differentiation by upregulation of Axin2 expression. In this study, PEMF stimulation decreased the expressions of Axin2 in GIOP rats. We speculated that PEMF stimulation activated canonical Wnt signaling pathway for preventing adipogenesis in BMSCs by suppressing the expression of adipogenic gene and eventually improved the lipid metabolism disorders of GIOP rats. Improvement of lipid metabolism disorders can prevent the development of osteoporosis to a certain extent.

GCs depress canonical Wnt signaling pathway through increasing the expression of Wnt-antagonists Dkk-1 and SOST in rodents and cell cultures [12]. Dkk-1 and SOST bind to LRP5/6 receptors to inactivate Wnt signaling. In this study, the expressions of Dkk-1 and SOST have not significantly increased in GIOP rats after DXMT injection was stopped; it may be due to GCs absence. PEMF stimulation effectively suppressed the expressions of Dkk-1 and slightly suppressed the expressions of SOST. SOST is almost exclusively expressed in osteocytes and late osteoblasts [21]. The relative higher expression of SOST in PEMF group may be due to the number of osteocytes and late osteoblasts were increased during PEMF stimulation. We speculated that PEMF stimulation activates canonical Wnt signaling pathway which may be partially via suppression of Dkk-1.

Adequate calcium and vitamin D supplementation is important for GIOP patients because GCs induce an overall negative calcium balance [12]. In this study, calcium supplement treatment can prevent bone loss to a certain extent in GIOP rats, perhaps because the calcium gluconate absorption rate of rats is better than humans. We found that the moderate prevention effect seemed to have nothing to do with canonical Wnt signaling pathway. In addition, hyperlipidemia was improved in GIOP rats after calcium supplement treatment for 12 weeks, and our results showed that calcium gluconate had a comparatively faint inhibition to the expressions of PPAR-*γ* and FABP4. Jensen et al. [38] demonstrated that high extracellular calcium plays a negative role in adipocyte differentiation. Parra et al. [39] found the antiobesity effect of dietary calcium in male mice. Calcium supplementation may have some potential benefits of overcoming lipid metabolism disorders in GIOP rats.

In summary, our study clearly demonstrated that GIOP rats still need effective antiosteoporosis therapy after GCs administration was stopped. PEMF stimulation can prevent bone loss and improve lipid metabolism disorders with no apparent side effects in GIOP rats. Wnt10b/LRP5/*β*-catenin signaling pathway played an important role during PEMF stimulation. Certainly, other signaling pathways may possibly be activated by PEMF, such as parathyroid hormone pathways and insulin-like growth factor. The influence of inactivation of canonical Wnt signaling pathway on bone formation and lipid metabolism during PEMFs stimulation needs further investigation. However, our study suggests that PEMF treatment may be a suitable therapeutic method for GIOP and it would offer some potential benefits for GIOP patients.

## Figures and Tables

**Table 1:** Primer sequences for real-time PCR analysis.

Gene ID	Gene	5′-3′	Sequence
315294	Wnt10b	Forward	CAGGCTTTGTGTGGAGTCATT
		Reverse	GAGGTTCTGGGCTGTAGTGG

293649	LRP5	Forward	GACCTGATGGGACTCAAAGC
		Reverse	GGGTGAAGAAGCACAGATGG

84353	*β*-catenin	Forward	CTTACGGCAATCAGGAAAGC
		Reverse	GACAGACAGCACCTTCAGCA

25341	OPG	Forward	TCAAGAATGCCACAGAA
		Reverse	GTCACGAAGCGGGTGTAGT

117516	RANKL	Forward	GGGAGCACTAAGAACTGGTCA
		Reverse	TTGGACACCTGGACGCTAAT

29134	Axin2	Forward	AGTCAGCAGAGGGACAGGAA
		Reverse	CTTGGAGTGCGTGGACACTA

293897	Dkk-1	Forward	TGACCACAGCCATTTACCTC
		Reverse	ACAGAGCCTTCTTGCCCTTT

80722	SOST	Forward	GAATGGTAGGTGCCAGGAGCA
		Reverse	TTAGGTAGGTGCCAGGAGCA

367218	Runx2	Forward	CCTCTGACTTCTGCCTCTGG
		Reverse	GATGAAATGCCTGGGAACTG

25664	PPAR-*γ*	Forward	CGGTTGATTTCTCCAGCATT
		Reverse	TCGCACTTTGGTATTCTTGG

24252	C/EBP*α*	Forward	AGTTGACCAGTGACAATGACCG
		Reverse	TCAGGCAGCTGGCGGAAGAT

79451	FABP4	Forward	CGACCACCATAAAGAGGAGAC
		Reverse	AAACCACCAAATCCCATCAA

24383	GAPDH	Forward	CAGGAGGCATTGCTGATGAT
		Reverse	GAAGGCTGGGGCTCATTT

**Table 2:** Bone mineral density (BMD) of each group before and after treatment (g/cm^2^).

Groups		Part							
		Head	Upper limb	Femur	Trunk	Rib	Pelvis	Spine	Whole body
Control group	Prior to treatment	0.237 ± 0.010	0.153 ± 0.032	0.142 ± 0.011	0.125 ± 0.009	0.113 ± 0.006	0.132 ± 0.017	0.132 ± 0.008	0.149 ± 0.005
	Posttreatment	0.243 ± 0.016	0.144 ± 0.026	0.156 ± 0.012^#O^	0.125 ± 0.008^#^	0.110 ± 0.007^#^	0.134 ± 0.017^#^	0.137 ± 0.009^#^	0.153 ± 0.007^#^

GIOP group	Prior to treatment	0.225 ± 0.015^*∗*^	0.131 ± 0.031	0.127 ± 0.008^*∗*^	0.110 ± 0.007^*∗*^	0.099 ± 0.012^*∗*^	0.113 ± 0.013^*∗*^	0.108 ± 0.010^*∗*^	0.132 ± 0.004^*∗*^
	Posttreatment	0.235 ± 0.021	0.123 ± 0.032	0.132 ± 0.011	0.114 ± 0.007	0.102 ± 0.007	0.117 ± 0.016	0.111 ± 0.015	0.135 ± 0.009

Calcium group	Prior to treatment	0.221 ± 0.010^*∗*^	0.147 ± 0.035	0.120 ± 0.013^*∗*^	0.106 ± 0.009^*∗*^	0.102 ± 0.012^*∗*^	0.104 ± 0.012^*∗*^	0.110 ± 0.010^*∗*^	0.131 ± 0.005^*∗*^
	Posttreatment	0.237 ± 0.024	0.119 ± 0.033	0.129 ± 0.019	0.113 ± 0.014	0.100 ± 0.008	0.123 ± 0.018^O^	0.125 ± 0.018^#O^	0.141 ± 0.013

PEMF group	Prior to treatment	0.219 ± 0.006^*∗*^	0.138 ± 0.009	0.120 ± 0.009^*∗*^	0.107 ± 0.010^*∗*^	0.100 ± 0.013^*∗*^	0.101 ± 0.015^*∗*^	0.110 ± 0.007^*∗*^	0.133 ± 0.006^*∗*^
	Posttreatment	0.234 ± 0.024	0.112 ± 0.035	0.133 ± 0.018^O^	0.117 ± 0.008^O^	0.104 ± 0.008	0.117 ± 0.018^O^	0.130 ± 0.013^#O^	0.148 ± 0.008^#O^

Compared with the control group before treatment, ^*∗*^ *P* < 0.05; compared with the GIOP group after treatment, ^#^ *P* < 0.05; compared with before treatment, ^O^ *P* < 0.05.

**Table 3:** Bone mineral content (BMC) of each group before and after treatment (g).

Groups		Part							
		Head	Upper limb	Femur	Trunk	Rib	Pelvis	Spine	Whole body
Control group	Prior to treatment	2.02 ± 0.23	0.49 ± 0.25	2.33 ± 1.00	3.82 ± 1.05	1.25 ± 0.49	1.40 ± 0.23	1.73 ± 0.49	8.19 ± 0.64
	Posttreatment	2.04 ± 0.28	0.49 ± 0.23	2.79 ± 0.69	3.43 ± 0.97^#^	1.23 ± 0.60^#^	1.35 ± 0.53	1.85 ± 0.75^#^	8.31 ± 0.95^#^

GIOP group	Prior to treatment	1.80 ± 0.13^*∗*^	0.39 ± 0.27	2.31 ± 0.73	2.60 ± 0.64^*∗*^	0.67 ± 0.32^*∗*^	1.15 ± 0.26^*∗*^	0.80 ± 0.25^*∗*^	7.21 ± 0.63^*∗*^
	Posttreatment	1.85 ± 0.18	0.34 ± 0.10	2.32 ± 0.69	2.32 ± 0.73	0.58 ± 0.31	1.10 ± 0.37	0.80 ± 0.28	7.06 ± 0.57

Calcium group	Prior to treatment	1.82 ± 0.10^*∗*^	0.42 ± 0.23	2.62 ± 1.01	2.12 ± 0.33^*∗*^	0.53 ± 0.11^*∗*^	1.00 ± 0.34^*∗*^	0.81 ± 0.25^*∗*^	7.14 ± 0.77^*∗*^
	Posttreatment	1.90 ± 0.32	0.35 ± 0.19	2.58 ± 0.65	2.44 ± 0.41	0.61 ± 0.20	1.24 ± 0.32^O^	0.91 ± 0.33	7.52 ± 0.71^O^

PEMF group	Prior to treatment	1.88 ± 0.16	0.53 ± 0.27	2.25 ± 0.97	2.27 ± 0.40^*∗*^	0.53 ± 0.12^*∗*^	0.95 ± 0.26^*∗*^	0.86 ± 0.32^*∗*^	7.09 ± 0.80^*∗*^
	Posttreatment	2.04 ± 0.21^O^	0.46 ± 0.23	2.73 ± 0.68	2.33 ± 0.39	0.63 ± 0.18^O^	1.02 ± 0.27	0.99 ± 0.34	7.68 ± 0.64^O^

Compared with the control group before treatment, ^*∗*^ *P* < 0.05; compared with the GIOP group after treatment, ^#^ *P* < 0.05; compared with before treatment, ^O^ *P* < 0.05.

**Table 4:** Histomorphometrical analysis of the fourth lumbar (L4) vertebral bodies.

Group	Tb.Ar (%)	Tb.Wi (*μ*m)	Tb.N (n/mm)	Tb.Sp (*μ*m)
Control group	54.23 ± 2.48	55.41 ± 5.69	9.85 ± 1.01	46.87 ± 6.29
GIOP group	26.95 ± 5.55^*∗*^	52.70 ± 4.67	5.25 ± 0.63^*∗*^	139.80 ± 25.68^*∗*^
Calcium group	42.69 ± 6.30^*∗*#^	62.23 ± 5.05^#^	6.88 ± 1.06^*∗*#^	85.32 ± 19.91^*∗*#^
PEMF group	48.16 ± 4.28^#^	61.42 ± 2.63	7.84 ± 0.52^*∗*#^	66.55 ± 9.64^#^

Data were expressed as mean ± SD.

Tb.Ar: trabecular area; Tb.Wi: trabecular width; Tb.N: trabecular number; Tb.Sp: trabecular separation.

Compared with the control group, ^*∗*^ *P* < 0.05; compared with the GIOP group, ^#^ *P* < 0.05.
